# Quantitative Detection of Shiga Toxins Directly from Stool Specimens of Patients Associated with an Outbreak of Enterohemorrhagic *Escherichia coli* in Japan—Quantitative Shiga toxin detection from stool during EHEC outbreak

**DOI:** 10.3390/toxins7104381

**Published:** 2015-10-27

**Authors:** Eiki Yamasaki, Masanori Watahiki, Junko Isobe, Tetsutaro Sata, G. Balakrish Nair, Hisao Kurazono

**Affiliations:** 1 Division of Food Hygiene, Department of Animal and Food Hygiene, Obihiro University of Agriculture and Veterinary Medicine, Nishi 2-11, Inada-cho, Obihiro 080-8555, Hokkaido, Japan; E-Mail: hkurazon@obihiro.ac.jp; 2 Toyama Institute of Health, Imizu, Toyama 939-0363, Japan; E-Mails: masanori.watahiki@pref.toyama.lg.jp (M.W.); junko.isobe@pref.toyama.lg.jp (J.I.); t-sata@rhythm.ocn.ne.jp (T.S.); 3 Translational Health Science and Technology Institute, Faridabad, Haryana 121001, India; E-Mail: nairgb@thsti.res.in

**Keywords:** Shiga toxin, Enterohemorrhagic *Escherichia coli* outbreak, rapid detection, bead enzyme-linked immunosorbent assay

## Abstract

Detection of Shiga toxins (Stx) is important for accurate diagnosis of Enterohemorrhagic *Escherichia coli* infection. In this study, we quantitatively analyzed Stx protein in nine patients’ stool during an outbreak that occurred in Japan. Highly sensitive immunoassay (bead enzyme-linked immunosorbent assay (bead-ELISA)) revealed that the concentrations of toxins in stool of patients ranged from 0.71 to 10.44 ng/mL for Stx1 and 2.75 to 51.61 ng/mL for Stx2. To our knowledge, this is the first report that reveals the range of Stx protein concentrations in human stools.

## 1. Introduction

A serious food-poisoning outbreak caused by Enterohemorrhagic *Escherichia coli* (EHEC) occurred from April to May 2011 in Japan. A total of 181 patients, including 21 patients with acute encephalopathy (AE) and five deaths among 34 patients with hemolytic-uremic syndrome (HUS) were recorded. The outbreak was caused by contaminated food at a barbecue restaurant chain in Toyama, Fukui, Ishikawa, and Kanagawa Prefectures. The epidemiological analysis revealed that raw beef dish called *yukhoe* was the contaminated food that caused the outbreak [1,2]. This outbreak had a tremendous impact on society, and led to change in government regulations of raw meat consumption in Japan.

In the outbreak, *shiga toxin gene* (*stx*)-negative *Escherichia coli* (*E. coli*) O111 were isolated from 52 cases in addition to 37 cases of *stx*-positive *E. coli* (*i.e.*, EHEC) O111, and EHEC O157 were isolated from 30 patients [3]. Although multiple serotypes were isolated from some patients, case-control study suggested that EHEC O111 might have played a primary role in causing severe symptoms [2,3]. Of the 37 EHEC O111-positive cases, 17 cases (45.9%) developed HUS, whereas of the 30 EHEC O157-positive cases, only nine (30.0%) developed HUS. In addition, within 16 patients they had an onset of HUS without EHEC isolation, 10 patients had anti-O111 antibody in their serum samples, whereas only two patients had anti-O157 antibody.

Shiga Toxin (Stx) 1 and 2 are the major toxins of EHEC. They cause endothelial damage with consecutive systemic thrombotic microangiopathy, resulting in hemorrhagic colitis and subsequent renal failure. The *stx1* and *stx2* genes belong to different but similar prophage genomes. The Stx-converting phages have been reported to be lost during *in vitro* culture [4], and are thought to be lost under *in vivo* conditions too. Genotyping based on pulsed-field gel electrophoresis (PFGE) and multilocus variable-number tandem-repeat analysis (MLVA) revealed that *stx*-negative *E. coli* O111 and EHEC O111 isolates in outbreak 2011 were closely related to each other. Based on these data, the *E. coli* O111 isolates with different *stx* gene profiles were presumed to originate from single clone. In addition, the PFGE and MLVA data indicated that all the EHEC O157 isolates that were confirmed in 30 patients might originate from a single clone although *stx* gene profiles were diverse (*stx_1_*, *stx_2_* and *stx_1_*/*stx_2_*) [3]. These data implicated that losses of *stx* genes presumably have occurred during the outbreak in 2011. This conclusion was supported by our previous study indicating that Stx-converting phage was easily lost during *in vitro* subculture of the EHEC O111 isolates which were isolated in the outbreak in 2011 [3].

Stx protein or *stx* gene detection directly from stool specimens without cultivation are attractive and efficacious methods for rapid diagnosis of EHEC infections, and the results obtained at such an early stage will be helpful for the effective management of the patients and would also assist in the prevention of the spread of the outbreak. On the other hand, the direct detection of Stx protein or *stx* gene is difficult [5]. In the case of *stx* genes detection, we have to be concerned about the possibility that Stx-converting phages can be easily lost during infections. Furthermore, in the case of Stx protein detection, especially by immunoassay, the amount of Stx in stools is often too low to detect [6]. In the present study, we successfully quantified the ranges of Stx proteins in stool specimen of patients in the food-poisoning outbreak by using a highly sensitive immunoassay (bead enzyme-linked immunosorbent assay (bead-ELISA)). Measurement of actual fecal Stx protein concentrations, which are easy to compare across independent sporadic/pandemic EHEC infections, is expected to provide useful information for epidemiological studies. In addition to epidemiological significance, it might contribute to construct appropriate *in vitro* and *in vivo* experimental designs because these experiments should be done with the range of toxin concentration that could be observed in specimens from patient.

## 2. Results and Discussion

**Figure 1:**
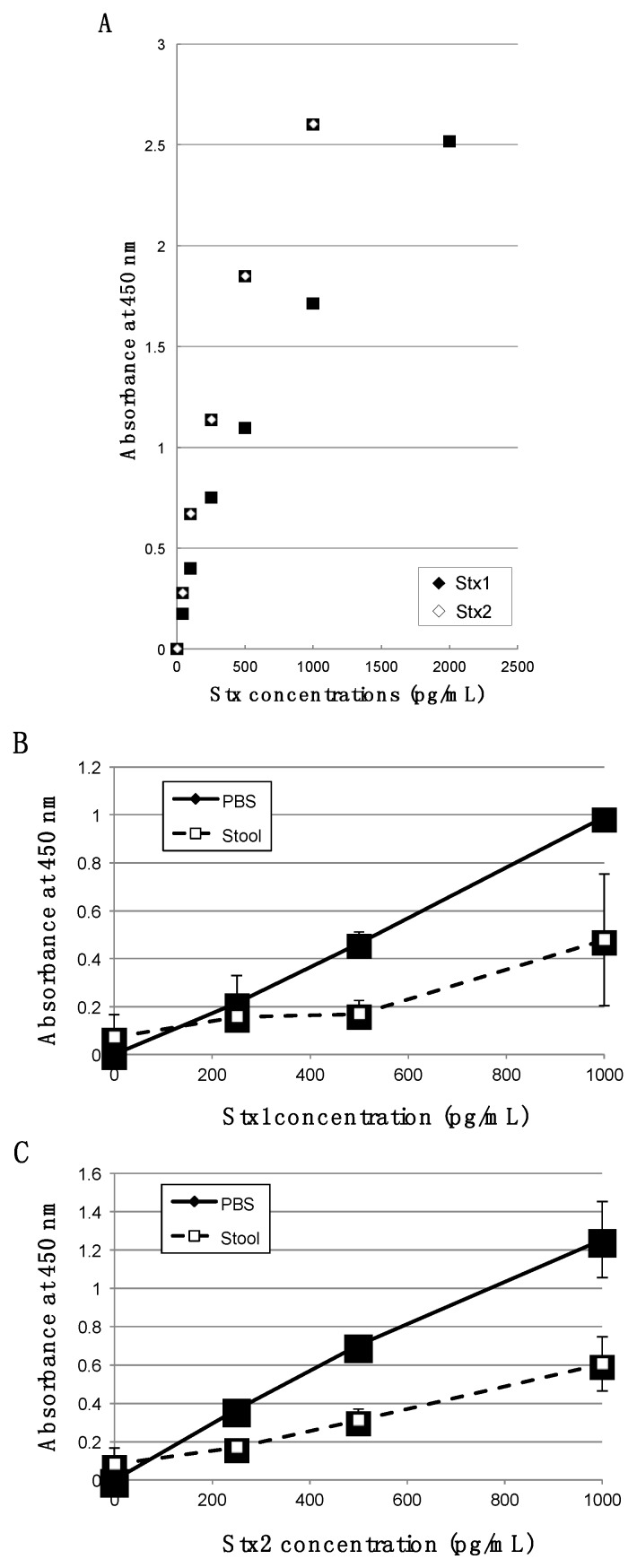
Detection abilities of bead-ELISA for Stx1 and Stx2: (**A**) Representative standard curve for Stx1 and Stx2 determined by bead-ELISA. Purified recombinant Stx1 or Stx2 at the concentration indicated were assayed by bead-ELISA for Stx1 (black rhombus) or Stx2 (white rhombus), respectively. Standard curves were independently prepared for each analysis. (**B**, **C**) Effect of stool specimen on Stx detections. Purified recombinant Stx1 (**B**) or Stx2 (**C**) suspended in PBS (black rhombus) or stool specimen obtained from healthy human (white square) were assayed by bead-ELISA for Stx1 (**B**) or Stx2 (**C**), respectively. Data are means ± SD of values from three experiments.

Bead-ELISA is an immunological detection system, which uses 6-mm-diameter polystyrene beads as the solid phase. Oku *et al.* developed bead-ELISA for detection of some bacterial toxin proteins including thermostable direct hemolysin of *Vibrio parahaemolyticus*, heat-labile enterotoxin of *E. coli*, cholera toxin and Stx [7]. The detection limits of these systems were as low as 6–200 pg/mL of the toxin. The standard curves for Stx1 and Stx2 by the bead-ELISA used in this study are shown in Figure 1A. The least Stx protein amounts that could be consistently detected were 250 pg/mL for purified recombinant Stx1 and 100 pg/mL for purified recombinant Stx2. We examined if Stx detection was affected when examined directly from stool specimens (Figure 1B,C). We observed some inhibitions of Stx detection in stool specimens.

We obtained nine stool specimens from nine individual patients affected by the outbreak in 2011. Sex, age and symptoms of the patients in addition to serotypes and *stx* genes profile of *E. coli* isolated from each patient’s stool are summarized in Table 1. For each stool specimens, we quantitatively-analyzed Stx1 and Stx2 proteins by using bead-ELISA as previously described [7,8,9]. In these assays, stool specimens were examined without any cultivation to reveal the actual concentration of toxins in stool specimens. Apparent concentration in patients’ stools obtained by bead-ELISA ranged from 0.71 to 10.44 ng/mL for Stx1 and 2.75 to 51.61 ng/mL for Stx2. Four out of nine samples examined were positive for Stx1 protein and four out of nine samples were positive for Stx2 protein. In total, five out of nine samples were positive for Stx1 and/or Stx2 proteins. Interestingly, in some cases, Stx protein profiles in stool specimens were not consistent with *stx* gene profiles of *E. coli* strain isolated from the same stool specimen [3]. In the case of patient No. 4, Stx protein could be detected, whereas EHEC was not isolated. No amplicon of *stx* gene had been detected directly from stool specimen of patient No. 4 either (data not shown), although internal control 16S rDNA, which was amplified with the universal primer for it, was successfully amplified. In contrast, in the case of patient No. 5, Stx protein could not be detected, whereas *stx* genes were detected in the *E. coli* isolates. Such differences between Stx protein profiles and gene profiles were also observed in some previous studies [10,11]. In addition, differences between fecal Stx profiles, anti-Stx antibodies profiles in patient serum and Stx profile on polymorphonuclear leukocytes has also been reported [10,12]. We considered that one of the reasons behind such differences is instability of Stx-converting phages. The instability of Stx-converting phages was suggested by the genetic homogeneity between *E. coli* isolates with different *stx* gene profiles as observed in our previous study [3]. These observations obviously suggest the importance of multiple analyses to carry out proper diagnosis of EHEC infections.

**Table 1:** Characteristics of patients, infecting isolates and Stx protein concentrations in stools.

Profiles of Patients				Profiles of *E. coli* Isolated from Stool *^,1^			Stx Protein Concentration in Stool (ng/mL)	
Patient ID	Sex	Age Group (years old)	Symptoms	Serotype	*Stx1* Gene	*Stx2* Gene	Stx1	Stx2
No. 1	Female	20–29	Bloody diarrhea, Abdominal pains, Vomit	O157:H7	+	-	10.44	2.75
				O157:H7	+	+		
				O111:H8	-	-		
No. 2	Female	40–49	Bloody diarrhea, Abdominal pains, HUS	O111:H8	-	-	U.D. *^,2^	U.D. *^,2^
No. 3	Male	10–19	Diarrhea, Abdominal pains, HUS	O157:H7	+	+	1.71	51.61
				O111:H8	-	+		
				O111:H8	-	-		
No. 4 *^,3^	Male	20–29	Bloody diarrhea, Abdominal pain, Vomit	N.D. *^,4^	n.d. *^,5^	n.d. *^,5^	0.71	U.D. *^,2^
No. 5	Female	0–9	Bloody diarrhea, Abdominal pains, HUS	O157:H7	+	+	U.D. *^,2^	U.D. *^,2^
				O111:H8	-	+		
				O111:H8	-	-		
No. 6	Male	10–19	Bloody diarrhea, Abdominal pains	O111:H8	-	+	U.D. *^,2^	12.88
No. 7	Female	10–19	Bloody diarrhea, Abdominal pains, Vomit	O157:H7	+	+	0.71	3.22
				O111:H8	-	+		
				O111:H8	-	-		
No. 8	Male	30–39	Bloody diarrhea	O111:H8	-	-	U.D. *^,2^	U.D. *^,2^
No. 9	Female	20–29	Diarrhea, Abdominal pains, Vomit, HUS, AE	N.D. *^,4^	n.d. *^,5^	n.d. *^,5^	U.D. *^,2^	U.D. *^,2^

*^,1^ Isolation and analysis of *E. coli* were performed as elaborated in our previous reports [1,2,3]. All the Stx1 and Stx2 from both the EHEC O111 and O157 isolates in this outbreak were subtype Stx1a and Stx2a, respectively [3]; *^,2^ Undetectable: Stx protein could not be detected; *^,3^ Patient No. 4 required dialysis treatment; *^,4^ Not detected: *E. coli* isolate had not been isolated from stool. *^,5^ Not determined: *stx* gene profile did not be examined because no *E. coli* was isolated.

The semi-quantitative Vero cell assay has been commonly employed to determine the amount of Stx in stool specimens [5]. Although the Vero cell assay has high sensitivity, it requires a couple of days to complete the analysis. Immunoassay is an alternative rapid methodology, while its sensitivity is usually lower than Vero cell assay [6]. Some commercial immunoassays are available, but most of them recommend cultivation of stool specimens before analysis [13,14]. If the cultivation were done before analysis, actual concentration of Stx in patient stools would not be known. In the present study, we performed the quantitative immunoassay without pre-cultivation of stool specimens. As a result, we achieved the actual concentration of Stx in patient stools. To our knowledge, this is the first report of determining the actual concentration of Stx in patients’ stools. Because some inhibition by control stool specimens was unfortunately observed in our bead-ELISA system (Figure 1B,C), we might not achieve the exact concentrations of Stx proteins in patients’ stools. Although apparent concentrations observed in this study may be an underestimate, information about range of Stx concentration in patient’s stool was revealed. We think the information about range of Stx concentrations might be useful because, for example, to construct appropriate experiment designs, both *in vitro* and *in vivo* studies should be done with the range of toxin concentrations that could be observed in specimens from patient. In addition, actual concentrations of Stx in stools are expected to facilitate cross-case (or cross-outbreak) comparison that might potentially gain new epidemiological knowledge of EHEC infection and outbreak, because, to carry out cross-case comparison, actual concentration is more convenient than relative concentration that is obtained by semi-quantitative assays.

## 3. Experimental Section

### 3.1. Patient Stool Specimens

The stool specimens were collected and stored at Toyama Institute of Health [1]. These specimens were stored at −30 °C until just before evaluation.

### 3.2. Preparation of Purified Recombinant Stx1 and Stx2

The laboratory stock of anti-Stx1 antiserum and anti-Stx2 antiserum were used for preparation of anti-Stx1 IgG conjugated column and anti-Stx2 IgG conjugated column. These antisera stocks were prepared according to a protocol previously described [7]. For antiserum conjugated column preparation, the laboratory stocks of antisera were coupled to HiTrap NHS-activated HP (5 mL, Amersham Biosciences, Buckinghamshire, UK) according to the manufacture’s instruction.

For Stx1 and Stx2 purification, previously constructed *E. coli* strain YO-01 (pKTJ5-15x) [15] and *E. coil* HB101 (pKTN817) strains [16] in which recombinant Stx1 and Stx2 were over expressed, respectively, were inoculated into Luria-Bertani (LB) broth supplemented with ampicillin (50 μg/mL). After over night cultivation at 37 °C, the bacterial cells were collected by centrifugation, resuspended in PBS, and then disrupted by sonication. The obtained cell sonicates were centrifuged to obtain soluble fractions. Proteins in the soluble fractions were precipitated with 80% (NH_4_)_2_SO_4_, resuspended in PBS, treated with RNase and DNase, and then applied to anti-Stx1 antisera conjugated column or anti-Stx2 antisera conjugated column. After the columns were washed with PBS, recombinant Stx1 and Stx2 proteins were eluted with 0.1 M glycine-HCl, pH 3.0. After dialysis, purified samples were condensed using Centricon YM-10 (Merck Millipore, Darmstadt, Germany). Purities of the samples were confirmed by SDS-PAGE and silver staining (Figure S1). Concentrations of the purified samples were determined by Bio-Rad Protein Assay Dye Reagent Concentrate (Bio-Rad, Hercules, CA, USA) using bovine serum albumin as a standard.

### 3.3. Preparation of Antisera against Purified Recombinant Stx1 and Stx2 and Construction of Bead-ELISA for Stx1 and Stx2 Detection

Antisera against purified recombinant Stx1 and Stx2 were raised in immunized rabbits according to the protocol previously described for other toxins [17]. Bead-ELISA were developed as described previously aside from the use of antisera against recombinant toxins [7]. The specificities of bead-ELISA were confirmed by the tests to various food-borne pathogens and other AB_5_ toxins (Figure S2).

### 3.4. Quantitative Analysis of Stx1 and Stx2 by Bead-ELISA

Stool specimens, for which no pretreatment such as filtration and incubation was done, were assayed directly for free fecal Stx1 and Stx2 by using the bead-ELISA as previously reported [7,9]. The stool specimens were diluted with Buffer A (0.01 M sodium phosphate buffer (pH7.0) containing 0.1 M NaCl, 0.1% bovine serum albumin and 0.1% NaN_3_) when they were not watery or the concentrations of Stx proteins in the specimens were too high. To determine the minimum detection ability of bead-ELISA used in this study and prepare standard curve for quantitative analysis, we performed the assay using various dilutions of purified recombinant Stx1 and Stx2 in PBS (Figure 1A) with the same procedure as for stool specimens. The 4 Parameter Logistic nonlinear regression model was used for curve-fitting analysis to determine fecal Stx1 and Stx2 concentrations. For the curve-fitting analysis, we used the standard curves prepared with Stx1 or Stx2 proteins diluted in PBS.

### 3.5. Ethical Considerations

This study was approved by the Ethical Review Board at the Obihiro University of Agriculture and Veterinary Medicine (approval No. 2012-02 and 2015-02) and Toyama Institute of Health (approval No. 2012-7).

## 4. Conclusions

When the diagnosis of EHEC infection is carried out, detection of Stx is indispensable. In this study, we suggested the importance of multiple analyses to carry out proper diagnosis of EHEC infection, because it has been revealed that there are differences between Stx protein profiles in stool specimens and *stx* gene profiles of *E. coli* isolated from the stool specimen. In addition, we revealed the range of fecal Stx protein concentrations of patients in food-born outbreaks. We expect that the information about fecal Stx protein concentrations can be helpful reference data not only for epidemiological analysis but also for *in vivo*/*in vitro* experiments.

## Supplementary Files

Supplementary File 1
