# Supplementary material for: Quantitative Detection of Shiga Toxins Directly from Stool Specimens of Patients Associated with an Outbreak of Enterohemorrhagic Escherichia coli in Japan—Quantitative Shiga toxin detection from stool during EHEC outbreak
Source: Toxins (Basel). 2015 Oct 27;7(10):4381–9. doi: 10.3390/toxins7104381 (PMC4626740; doi:10.3390/toxins7104381)
Supplement: Supplementary File 1 [file toxins-07-04381-s001.pdf]

Supplementary Materials

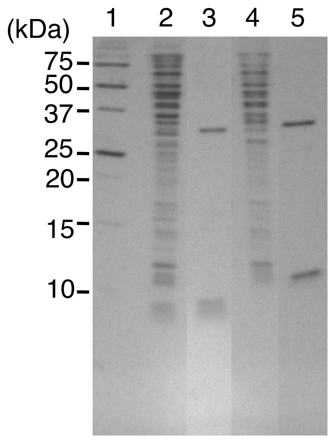

Figure S1. Purities of purified recombinant Stx1 and Stx2 proteins. Purity of purified recombinant Stx1 and Stx2 were examined by SDS-PAGE followed by silver staining. Purified preparations gave two stained bands correspond to A and B subunits of Stx proteins. 1: molecular weight maker; 2: whole cell lysate of *E. coli* strain YO-01 (pKTJ5-15x); 3: purified recombinant Stx1 (625 ng); 4: whole cell lysate of *E. coil* HB101 (pKTN817) strain; 5: purified recombinant Stx2 (625 ng).

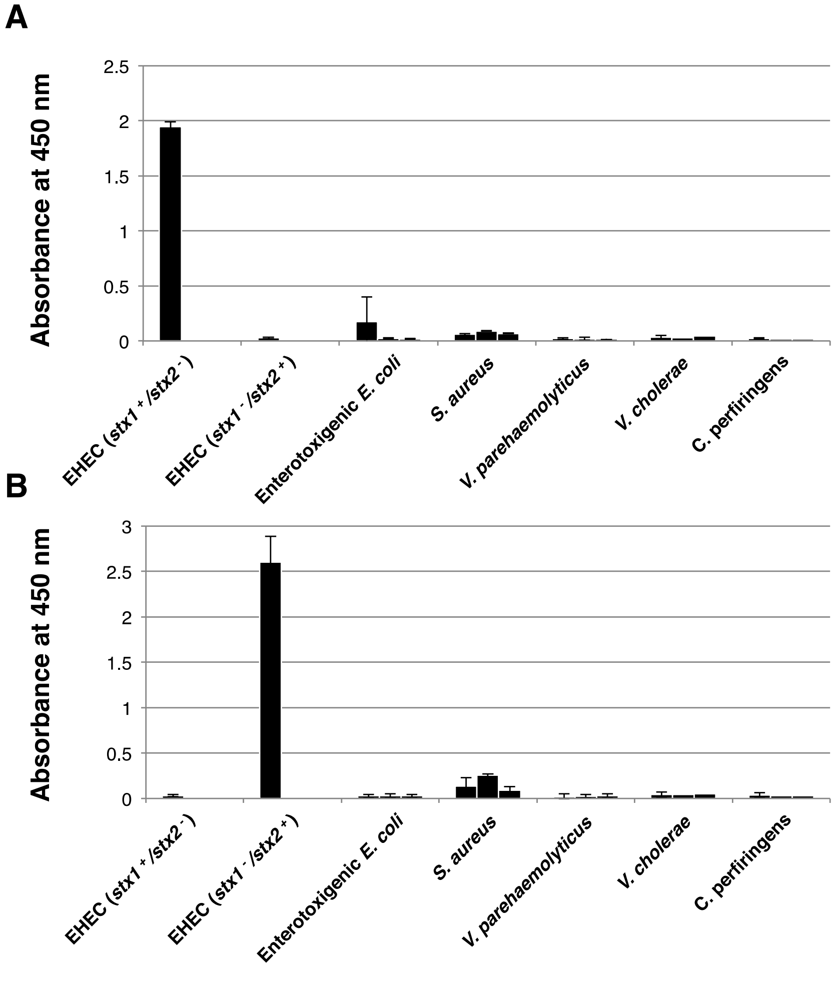

Figure S2. *Cont.*

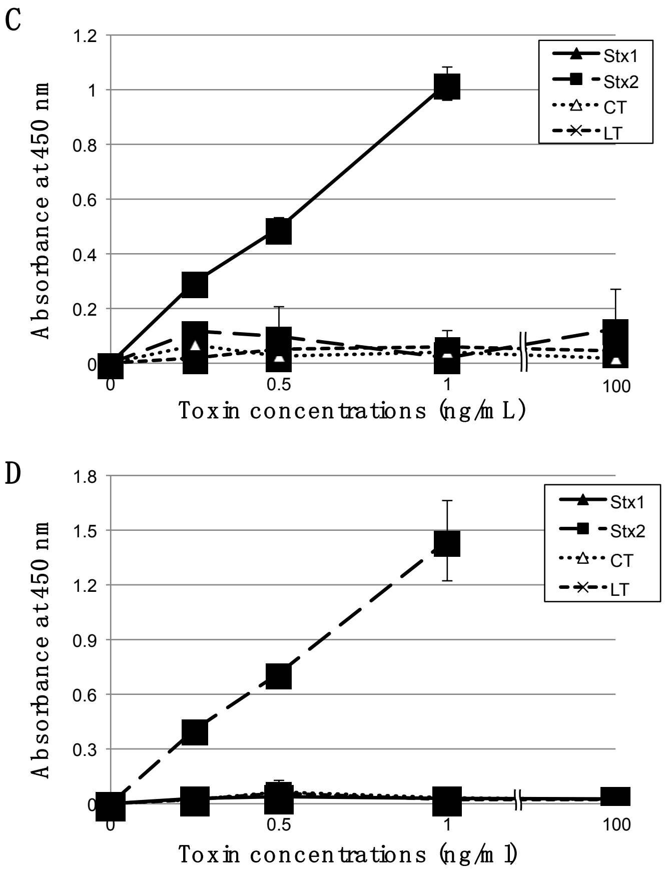

Figure S2. Specificities of bead-ELISA. (**A**,**B**) Various food-borne pathogens were examined by bead-ELISA specific for Stx1 (**A**) and Stx2 (**B**). Overnight cultures of EHEC (in CAYE broth), Enterotoxigenic *E. coli* (in CAYE broth), *S. aureus* (in BHI broth), *V. parahaemolyticus* (in Mannitol-salt peptone water), *V. cholerae* (in AKI broth) and *C. perfringens* (in Duncan-Strong Broth) were sonicated with maximum power of Handy Sonic UR-20P (TOMY SEIKO CO., LTD, Tokyo, Japan) for 45 s. Clear fractions obtained after centrifugation (12,000× *g* for 10 min.) of sonicated cultures were analyzed by bead-ELISA. For Enterotoxigenic *E. coli*, *S. aureus*, *V. parahaemolyticus*, *V. cholera* and *C. perfringens*, 3 independent isolates were analyzed, and each bars indicates the results for each independent isolates. Data are means ± SD of values from three experiments. No nonspecific reactions were observed in all the experiments; (**C**,**D**): Purified AB_5 toxins were examined by bead-ELISA specific for Stx1 (**C**) and Stx2 (**D**). Various concentrations of purified recombinant Stx1, Stx2, cholera toxin (CT) and *E. coli* heat-labile enterotoxin (LT) were examined by bead-ELISA. Data are means ± SD of values from three experiments. No nonspecific reactions were observed in all the experiments.
